# External control arms: COVID-19 reveals the merits of using real world evidence in real-time for clinical and public health investigations

**DOI:** 10.3389/fmed.2023.1198088

**Published:** 2023-07-06

**Authors:** Patrick Silva, Nora Janjan, Kenneth S. Ramos, George Udeani, Lixian Zhong, Marcia G. Ory, Matthew Lee Smith

**Affiliations:** ^1^Institute of Bioscience and Technology and Department of Translational Medical Sciences, College Station, TX, United States; ^2^Center for Community Health and Aging, School of Public Health, Texas A&M University, College Station, TX, United States; ^3^Department of Clinical Pharmacy, School of Pharmacy, Texas A&M University, College Station, TX, United States; ^4^Department of Pharmaceutical Sciences, School of Pharmacy, Texas A&M University, College Station, TX, United States

**Keywords:** external control arm, real world data, regulatory science, pharmacogenomics, oncology, pragmatic study

## Abstract

Randomized controlled trials are considered the ‘gold standard’ to reduce bias by randomizing patients to an experimental intervention, versus placebo or standard of care cohort. There are inherent challenges to enrolling a standard of care or cohorts: costs, site engagement logistics, socioeconomic variability, patient willingness, ethics of placebo interventions, cannibalizing the treatment arm population, and extending study duration. The COVID-19 pandemic has magnified aspects of constraints in trial recruitment and logistics, spurring innovative approaches to reducing trial sizes, accelerating trial accrual while preserving statistical rigor. Using data from medical records and databases allows for construction of external control arms that reduce the costs of an external control arm (ECA) randomized to standard of care. Simultaneously examining covariates of the clinical outcomes in ECAs that are being measured in the interventional arm can be particularly useful in phase 2 trials to better understand social and genetic determinants of clinical outcomes that might inform pivotal trial design. The FDA and EMA have promulgated a number of publicly available guidance documents and qualification reports that inform the use of this regulatory science tool to streamline clinical development, of phase 4 surveillance, and policy aspects of clinical outcomes research. Availability and quality of real-world data (RWD) are a prevalent impediment to the use of ECAs given such data is not collected with the rigor and deliberateness that characterizes prospective interventional control arm data. Conversely, in the case of contemporary control arms, a clinical trial outcome can be compared to a contemporary standard of care in cases where the standard of care is evolving at a fast pace, such as the use of checkpoint inhibitors in cancer care. Innovative statistical methods are an essential aspect of an ECA strategy and regulatory paths for these innovative approaches have been navigated, qualified, and in some cases published.

## 1. Introduction

Randomized controlled trials (RCTs) have been considered the ‘gold standard’ in clinical research, despite recent concerns about external generalizability and feasibility (1–3). Traditionally using 1:1 randomization to assign patients into the experimental (i.e., study intervention) and control (i.e., standard of care) study arms, RCTs often take years to complete. Further, RCTs are often impractical study designs for rare diseases. Generalizability can be limited by RCT enrollment criteria that often excludes common comorbidities. While multi-institutional trials are intended to overcome the inefficiencies, recruitment challenges, and costs of RCTs within an institution, these multi- institutional trials often do not expand the eligibility criteria of the Phase II efficacy trial to make the study more generalizable (4).

Clinical trial models have evolved to improve the sensitivity and efficiency of analysis. Reducing the overall number of study participants needed to evaluate trial outcomes statistically places fewer patients at risk for harm and reduces the number of patients randomized to a potentially less effective therapeutic control arm. The size of the intervention arm and the size of the control arm have different ethical and practical considerations but reducing each can have benefits to patients and study sponsors. In rare diseases, a control arm may be impractical, unethical, or otherwise impossible (5). Batten disease, a rare fatal inherited disorder, is a type of neuronal ceroid lipofuscinosis, in which the nervous system is unable to recycle certain degradation products. In clinical trial designs, the choice of a control arm is an especially critical aspect of trial design in which ethical and scientific issues are deeply entwined (6). A clinical trial for cerliponase alfa faced the challenge of very few patients available to enroll, and an ethical quandary about the best comparator to show efficacy with scientific rigor. The only practical control was to compare to disease progression in a historical standard of care cohort (7) using a curated global registry enabled this approach to clinical validation with cerliponase alfa (8).

The purposes of this paper are to: (1) highlight innovative statistical methods and document methodological challenges with clinical trials conducted during the COVID-19 pandemic in the US and internationally; and (2) offer recommendations for alternative approaches to capture real world data and utilize external control arms in clinical trials.

## 2. Clinical trials during the COVID-19 pandemic

### 2.1. Changing best practices

Long-awaited reforms to generally accepted best practices for clinical trials were underscored during the COVID-19 pandemic. Using global technological advances, clinical outcomes were rapidly shared, often outside of formal clinical trials, due to the dire need to support patients and understand the pathophysiology of the disease. In contrast to the rapid identification and development of therapeutics and vaccine development for COVID-19, traditional development of therapeutics is highly inefficient before clinical validation and deployment is reached (9). This was particularly evident during the COVID-19 pandemic when the ethics of therapeutic randomization were questioned because best supportive care was ill-defined and rapidly changing (10). Many generally accepted clinical trial practices, were at odds with the immediate medical imperatives, and had to be reconsidered during the COVID-19 pandemic. Specifically for oncology, there was a call to rethink clinical trial dogma and revamp clinical trial design to increase efficiency, avoid highly restrictive eligibility criteria, and address the clinical needs of patients (11–13).

### 2.2. Limitations in traditional RCT approaches

Multiple attempts were made at conducting RCTs to validate therapeutics during the COVID-19 pandemic illustrating limitations in traditional RCT approaches. We provide several examples highlighting challenges in interpretation of findings:

(i) For example, patients (*n* = 596) with moderate COVID-19 presentations (defined as having pulmonary infiltrates and room-air oxygen saturation of >94%) were enrolled in a study from March 15 through April 18, 2020 at 105 hospitals in the United States, Europe, and Asia (14). Patients were randomized to receive remdesivir or standard care. Using a 1:1:1 ratio, patients either received a 10- day course of remdesivir (*n* = 197), a 5-day course of remdesivir (*n* = 199), or standard care (*n* = 200). Four months later, the published results showed no statistically significant difference between a 10- day course of remdesivir and standard care. While a 5-day course of remdesivir resulted in a statistically significant improvement in status, the difference was of “uncertain clinical importance.” There were significant study limitations as hospital discharge rates varied greatly across regions, the open-label design potentially led to bias, some laboratory parameters were not collected, and viral loads were not assessed (15). This illustrates inherent challenge of achieving a perfect comparator group in a RCT. Put another way, an RCT is theoretically ideal but sometimes an imperfect and impractical assessment of a therapeutic in a real-world setting.

(ii) In another example, retrospective meta-analysis was performed using pooled data from 7 RCTs, conducted from February 26, 2020 to June 9, 2020, in 12 countries that evaluated the efficacy of three corticosteroid regimens in the treatment of COVID-19. The three corticosteroid regimens were systemic dexamethasone (3 trials, 1,282 patients, and 527 deaths), hydrocortisone (3 trials, 374 patients, and 94 deaths), and methylprednisolone (1 trial, 47 patients, and 26 deaths). The study included a total of 1,703 critically ill COVID-19 patients out of the 1,920 patients that were planned for statistical analysis. There was little inconsistency between individual study outcomes, resulting in few differences observed in the meta-analysis. All studies showed that systemic corticosteroids significantly lowered the 28-day all-cause mortality compared with usual care or placebo. The odds ratio (OR) for mortality with dexamethasone was 0.64 (95% CI, 0.50–0.82; *p* < 0.001), hydrocortisone was 0.69 (95% CI, 0.43–01.12; *p* = 0.13), and methylprednisolone was 0.91 (95% CI, 0.29–2.87; *p* = 0.87) (14).

(iii) In another case, an attempt was made to conduct a multicenter randomized double- blinded sequential trial in France that evaluated the effectiveness of hydrocortisone in COVID-19 intensive care unit (ICU) patients with respiratory failure. The primary outcome measure, treatment failure on day 21, was defined as death or persistent dependence on a ventilator or high-flow oxygen therapy. The study began March 7, 2020, and was stopped on June 29, 2020 when an interim analysis was performed. Although likely underpowered to identify a statistically and clinically important difference in the study endpoint, the trial was stopped early after 149 of the 290 patients were enrolled based on the strength of the interim results of this and other studies (16).

### 2.3. Studies evaluating quality of RCTs

We also note two studies explicitly evaluating the quality of randomized clinical trials. The first is a survey of the 516 COVID-19 randomized clinical trials, registered in ClinicalTrials.gov and the World Health Organization International Clinical Trials Registry Platform between January 1 and April 9, 2020, conducted approximately a year later in October 2020. The survey evaluated whether the trials recruited 75% or more of their target sample size, stopped before reaching 75% recruitment, or continued to recruit patients but had not yet met the 75% accrual (either on schedule or delayed). Of the 516 randomized trials, only 53 (10.3%) of the 56 completed studies had been published (30%), had not started or were discontinued, and 24 (4.6%) were terminated early. The remaining studies were ongoing but only 126 (24.4%) were on schedule. Of the RCTs initiated in the first 100 days of the pandemic, 30% did not begin recruitment and only 10% had results reported by mid- October 2020. Of the 24 terminated and 46 discontinued trials, 14 RCT investigators communicated that the trials were discontinued due to decreasing COVID-19 cases (8 trials [57.1%]), emerging data regarding safety (6 trials [42.9%]), or futility (2 trials [14.3%]). Importantly, the statistically significant safety concerns, affirmed through peer-review, were derived from observational studies before a RCT could be completed (17). A high rate of multiplicity was noted during a June 8, 2020, review of the COVID-19 trials on ClinicalTrials.gov. This multiplicity was thought to enhance the likelihood of finding a positive result through chance alone resulting in the widespread administration of a potentially ineffective therapeutic. Fragmentation of efforts also lead to competition for study participants that compromised clinical trial accrual and the statistical power of all trials (18).

Second, a cross-sectional analysis was conducted of the characteristics and the strength of evidence of COVID-19 studies registered on ClinicalTrials.gov on May 19, 2020. There were 640 observational and 664 RCTs. Over 75% (*n* = 1,180) of the RCTs and observational COVID-19 studies were conducted at a single center, and only 29.1% of the COVID-19 studies (RCTs and observational) could potentially yield the OCEBM level 2 evidence, or the highest level of evidence (19). Among the RCTs, only 35.8% of studies had planned enrollment of more than 100 participants, and 17% involved at least 2 study centers, which is required for the highest level of evidence. Also, to fulfill the highest level of evidence criteria, only 29.1% of the RCTs were placebo-controlled, and only 11.3% of the placebo-controlled RCTs were blinded and conducted in at least two centers. Among the observational studies, 80.8% were conducted in a single center, and only 13.6% were prospective cohort studies that could yield the highest level of evidence (19).

### 2.4. Observational and therapeutic reports

By necessity, many of the COVID-19 studies were observational and single-arm therapeutic reports. These studies followed the Strengthening the Reporting of Observational Studies in Epidemiology (STROBE) Guideline for Cohort Studies. Research consortia were also developed to evaluate the variabilities in therapeutic approaches, and to pool sufficient patient numbers for statistical analysis. An observational study of 2,483 consecutive admissions for confirmed COVID-19 was conducted from a 5-hospital health system. The outcomes of remdesivir (administered with or without a corticosteroid) were compared to matched-COVID-19 patients who did not receive remdesivir; matching was performed using time-invariant covariates and time-dependent covariates. From this study, remdesivir alone was associated with faster clinical improvement as evidenced by a 2-day shorter time to clinical improvement (adjusted hazard ratio 1.47; 95% CI 1.22–1.79) and a 7.7% (vs. 14.0%) 28-day mortality rate (adjusted hazard ratio 0.70; 95% CI 0.38–1.28) compared to matched controls (20). During the period of highest COVID-19-related mortality, from March 7, 2020, to June 17, 2020, a voluntary statewide collaborative initiative was established among 41% of the 92 noncritical access- nonfederal hospitals in Michigan to evaluate adherence to venous thromboembolism (VTE) anticoagulation regimens. Some of the hospitals included all COVID-19 patients, while high-volume hospitals used a pseudo-random sampling process to select cases given the limited availability of data abstraction resources. Pseudo-randomization, resulting from logistical constraints, involved evaluating all the potentially eligible patients; each day, one patient was selected for the study out of many eligible study patients based solely upon the time of hospital discharge (21).

## 3. Alternative approaches for external control arms

### 3.1. Real world data

In the best of circumstances, RCTs encounter difficulties in efficiently fulfilling study enrollment. During the restrictions and stressors of the COVID-19 pandemic, conducting and completing a RCT was made even more difficult. Providing information regarding the transmission and treatment of a novel virus, single-arm trials and the analysis of Real World Data (RWD) made significant scientific contributions to understanding the epidemiology of severe disease, and the efficacy of an evolving therapeutic model during the COVID-19 pandemic.

When a RCT is not feasible for either rare diseases or ethical reasons, historical or external control arms, derived from RWD, have been used (22). Additionally, historical or external control arms often are more accurate comparators as RCTs are not perfectly representative of adherence and public health factors affecting how a therapy may be used in the real world. A combination of regulatory policy shifts, legislation, clinical data availability, and next-generation sequencing have catalyzed the feasibility of ECAs (23). Using the dynamic borrowing model, in which a historical or RWD control group closely matches a small concurrent control group in the study, there is minimal impact on the final study conclusions. This minimal impact is maintained when up to 80% of a trial’s control group consists of closely matched historical or RWD controls (22).

### 3.2. Regulatory guidance

In December 2016, Section 3022 of the 21st Century America Cures Act was enacted and directed the U.S. Food and Drug Administration (FDA) to accept statistical methods that include RWD and Real World Evidence (RWE) (23). The FDA’s Center for Devices and Radiological Health (CDRH) and Center for Biologics Evaluation and Research (CBER) issued guidance on August 31, 2017 regarding the submission of RWD and RWE to the FDA (24, 25). To improve the regulation of combination products, the Cures Act also directed the FDA to create inter-center institutes to coordinate initiatives in the development of drugs, biologics, and devices. The Oncology Center of Excellence was the first inter-center institute created.

After the FDA guidance was issued, the CDRH reported that the use of RWE in premarket and post- market regulatory decisions increased by 193% compared to the 2015 baseline (26). However, an RCT was the statistical model used for approximately 60% of the studies investigating new therapeutic agents/devices (27). Given the inherent *concerns* for the safe administration of an agent/device, the enrolled clinical trial population may not be representative of the real world patient populations in which the agent is intended to be administered (Table 1). In general, the patient populations within RCTs are younger, more homogenous, and have fewer comorbidities than the actual patient populations (28, 29). Nearly 80% of clinical trials fail to meet the initial enrollment projection, and less than 10% of new therapeutic agents receive FDA commercial approval (30–33). RCTs may not always be representative of safety and efficacy within real world populations due to numerous and overlapping public health factors such as adherence, socioeconomic, genetics, polypharmacy and polychronic disease factors. Even after FDA approval based on an RCT, the marketing approval may be revoked or the FDA label changed (i.e., black box warnings) may occur because of unexpected adverse events within a real world population (34).

**Table 1:** Practical pros and cons of external control groups.

	RCT control groups		External control groups		
	Single institution	Multi-institution	Historical	Traditional	Contemporaneous
Allow for changes in standards of care	N	N	P	Y	Y
Decreased patient number required for clinical trial	N	N	Y	Y	Y
Ability to use digital twins	P	P	Y	Y	Y
Decreased time required for clinical trial	N	N	Y	Y	Y
Control arms receive current standard of care	Y	Y	P	Y	Y
Ethical concerns regarding inferior care in control arm	P	P	N	N	N
Receive treatment in real world healthcare setting	P	P	Y	Y	Y
Interim analysis possible	Y	Y	N	N	Y
Acceptance by FDA	Y	Y	Y	Y	Y
Increased time in getting novel treatments to clinic	Y	Y	N	N	N

Y represents-yes; N represents-no; P represents-perhaps.

RWD and RWE have 4 roles as defined by regulatory guidance. In the first role, the FDA uses RWD and RWE to monitor post-market safety (adverse events) and efficacy that resulted in regulatory decisions. Second, to expedite approval of innovative therapeutic approaches, RWD and RWE support clinical trial designs, including approval of new indications for approved drugs. Third, RWD and RWE are used to: (a) develop therapeutic guidelines and decision support tools for clinical practice; and (b) to support healthcare insurance coverage and reimbursement decisions. Specific examples include: (a) generating and refining hypotheses to be tested in a prospective clinical study; (b) identify, demonstrate, or support the clinical validity of a biomarker; (c) support label expansion for approved drugs; and (d) for public health surveillance and policy efforts (17, 18, 35).

The sources of RWD are broad and incorporate both governmental and commercial data sources. Accepted sources of RWD include electronic health records (EHRs), claims and billings databases, product and disease registries, patient-generated data, and health status data gathered from other sources, including mobile devices. The analysis of RWD provides information about, or enables comparisons of, the clinical outcomes associated with a therapeutic intervention and characterizes standards-of-care. These therapeutic outcomes generate RWE from a wide range of study designs that include data mining, observational, and interventional studies.

To improve access to innovative medical devices, the FDA formally approved the use of RWD and RWE to reduce premarket data collection, and to monitor safety and efficacy in the post-market setting (36, 37). As in all clinical investigations, the FDA requires that all data be reliable, accurate, and verifiable with robust quality control. RWD is also required to be large enough to evaluate the medical product in the specific regulatory context. Three determining factors for the use of RWD are whether: (1) sufficient patient numbers and detail is available for analysis; (2) confounding factors can be addressed; and (3) the database is generalizable to the involved population. For example, RWD may need to account for the off-label use of an agent.

In clinical trials, reliable and relevant RWD can be applied when traditional prospective data collection models are impractical, and ethical issues arise regarding treatment assignment (38–40). The FDA guidance emphasizes the role of external controls derived from RWD in the regulatory evaluation of medical devices, and for rare or life-threatening disease (41–44). The guidance also states that randomization does not guarantee the absence of bias; rather, it mitigates the known sources of bias. Multiple international agencies and academic groups have also advocated for and accepted non-randomized evidence within clinical trials (45, 46). The use of RWE has expanded along with next-generation sequencing (NGS)-based testing and other forms of high-content bioanalysis (47, 48). The population paradox is defined as understanding the clinical relevance of molecular differences within a population. The population paradox enables the use of clinicogenomics databases for therapeutic development, clinical trials design, and therapeutic decision making for individualized medical care (49–52). Evidence gaps, however, remain for the use of NGS with RWE in health insurance coverage decision-making with the exception of pharmacogenomics testing, suspected pediatric genetic disorders, and oncology (48).

To mitigate potential bias, the use of RWD also requires a well-defined study design, clinical endpoints, bias mitigation strategy, and statistical analysis that is comparable to traditional RCTs (53, 54). Especially when multiple data sources are used, a more diverse patient population may be possible. However, the data source must be reliable, and the data should be anonymized to protect the privacy of the patient privacy. If patient-specific data is needed, such as a biomarker result, the data can be tokenized through an intermediary to link the result to the anonymous data point. When RWE is submitted to the FDA, both the RWD and RWE must conform to recognized data standards for file formats and data structures using standardized variables and definitions (55).

### 3.3. External control arm

Medical paradigms are changing. The intended use and labeling of therapeutics are increasingly agnostic of medical subspecialty, target tissue or organ, or pathologic characterization (56). Inter- connecting biological pathways now define risks for the development of disease or a disease process, and the corresponding therapeutic strategy. Relevant outcomes are expanding beyond survival (57, 58). Instead, outcomes of interest now reflect the importance of controlling the symptoms and trajectory of the disease (58), quality of life indicators, and/or downstream healthcare utilization (59).

Greater emphasis is also placed on outcomes that occur after completion of the clinical trial and in real world populations (60). Real world outcomes can be used to better estimate the cost-of-illness from both the patient and societal perspective (61). Innovative statistical approaches are increasingly accepted within and outside the construct of RCTs, resulting in an evolution in the design and efficiency of conducting clinical trials (62). External control arms (ECAs), derived from RWD, are increasingly used to reduce the number of patients and time required for study completion (5). ECAs and historical control arms have been used for decades to evaluate new therapeutics in rare diseases (63). Most importantly, from an ethical perspective, ECAs also place fewer patients within a clinical trial at risk for adverse events and potentially limited therapeutic benefit (64).

### 3.4. Innovative statistical methods

Statistical methods have been developed to minimize the number of patients randomized to a control arm, while retaining sufficient power for primary endpoints and safety evaluations. Inefficiencies in conducting clinical trials often slow the availability of breakthrough therapies for patients. Chapple and Thall (65) introduced a novel and more efficient semi-parametric stochastic ordering (SPSO) model for Phase I-II trials. This new statistical model uses a flexible monotone increasing the toxicity model and a semi-parametric stochastic ordering model for efficacy probabilities. A novel sensitivity analysis of the range of correlations between toxicity and efficacy was then performed. As a previously unreported finding, they found that all prospective clinical study designs performed worse when there was a negative correlation between toxicity and efficacy.

Digital twins are commonly used in industry to build predictive models for clinical trial performance and the real-world implementation of a new therapy. The approach involves developing a statistical model using external data from a patient population that can predict the trajectory of the disease for a standard of care or control arm. Reducing the number of patients accessioned to the study by 30–50%, that standard of care arm is then used as a synthetic control that is compared to a single-armed interventional clinical trial. Comprehensive forecasting of Alzheimer’s Disease progression was accomplished using the unsupervised Conditional Restricted Boltzmann Machine (CRBM) learning model that incorporated 44 clinical variables in 1909 patients with mild manifestations of disease. The generated synthetic patient data, including the Alzheimer’s Disease Assessment Scale-Cognitive Subscale (ADAS-Cog) score, accurately reflected the means, standard deviations, and correlations of each variable over time. The accuracy of the Alzheimer’s Disease trajectory from the actual data could not be distinguished from the synthetic control by a logistic regression (66). Biological markers are used with greater frequency to personalize therapeutics and reduce risk from futile care. In oncology an ECA, derived from a de-identified clinico-genomic database, closely replicated the control arm from the randomized IMblaze370 study of metastatic colorectal cancer (67, 68). Such databases can not only match patients within the control arm but, potentially more important, may also more closely match patients randomized to the therapeutic arm of an RCT.

Confounders, such as comorbidities, may limit the generalizability of RCT results to the population identified within the FDA approved product label. Once FDA approved, unless specifically excluded in the FDA product label, a confounding factor may result in significant unanticipated adverse events that could outweigh the derived therapeutic benefit. The use of ECAs could support subsequent additional prospective single-arm Phase II-III studies that have a more expansive eligibility criteria in a limited patient cohort (69, 70). Substantially reducing the number of patients at risk, these prospective studies could provide critical toxicity and efficacy information more rapidly than a RWE retrospective review of large populations. This more rapid evaluation of relevant populations might find therapeutic benefit to a patient subpopulation that was not included in the foundational RCT, and/or prevent adverse events by identifying previously unknown risk factors. Using pharmacogenomic registry data, the genetic and pharmacologic co-variants of efficacy, safety and tolerability could also be determined (71). Known variants of CYP2C19 and CYP2D6 (72, 73), emergent α36 variants of ER (74), and drug–drug interactions with serotonin reuptake inhibitors (75) all have clinically relevant impacts on outcomes of breast cancer patients treated with tamoxifen. Covariates, that influence clinical outcomes, can be identified in clinical trials that use ECAs and a relatively small number of patients. Identifying covariates within product registration and Phase IV clinical trial designs can improve overall response and reduce toxicity rates.

External control arms and single-arm trials are increasingly accepted by regulators for drug approval, and Health Technology Assessment (HTA) bodies (63). ECAs have also been used to provide clinical context. A 13-fold increase in the use of ECAs was observed among the 433 single-arm trial submissions between 2011 and 2019 (76). Between 2015 and 2019, the use of ECAs increased 22%, and approximately half (52%; 226/433) of the submissions included ECAs (76). The ECAs included historical controls from prior clinical trials (24%; 104), and from RWD (20%; 87), while 40% (175) of single-arm trials did not incorporate an ECA (76). The overall acceptance rate for single-arm trial submissions was 48%, increasing to 59% with RWD ECAs. The acceptance rate of single-arm trials increased from 41% from 2015 to 2017 to 61% in 2018–2019 (76). Between 2015 and 2019, the acceptance rate for single- arm trials with historical controls as ECAs decreased by 10%, and with no ECAs decreased by 329 (16%) (54).

A proof-of concept study showed that the control arms in RCTs could be replicated by ECAs from curated electronic health record data. Applying study eligibility criteria, 9 advanced non-small-cell lung cancer trials were evaluated. Key aspects of the trials ranged from biomarker availability, study start dates, and overall survival as an endpoint. A comparison of the log hazard ratios among all RCTs and ECAs resulted in a 0.86 Pearson correlation coefficient (77). As an example, advanced non-small-cell lung cancer trials have used ECAs derived from de-identified contemporaneous electronic health records to determine overall survival within single-arm trials.

### 3.5. Contemporaneous external control arm

External control arms are useful as a comparator to validate the control arm of a RCT by revealing potential biases inherent in the study environment or enrolled population. Within an RCT, the goal is for the control group to mirror the experimental arm cohort as much as possible. With restricted study eligibility criteria, the RCT control and experimental arms may be matched to each other in terms of demographics and comorbidities, but these cohorts may not always be representative of the real- world patient population. Variations in outcomes may occur based on access to care, insurance payer, practice patterns, and health-system resources. Indeed, zip code is a major determinant of clinical trial variations in recruitment (78) and health outcomes (79). RCTs conducted within a single institution in a single location, versus a multi-Institutional study, will generally not account for population diversity. Diversity in outcomes, especially related to healthcare resource utilization and cost, are often attributable to the provider, payer and/or population factors. For example, many factors differ between academic medical centers in large urban areas vs. community oncology clinics, including health system resources and the social determinants of health.

As a specific illustration, outcomes were compared among de-identified patients with advanced non- small-cell lung cancer treated with programmed death 1/programmed death-ligand 1 inhibitors. The comparison involved RWD from an insurance claims database and EHRs obtained from 6 healthcare organizations (70). The datasets ranged from 269 to 6,924 patients. Correlations between real world time-to-treatment-discontinuation (rwTTD), time-to-next-treatment, and overall survival (rwOS) ranged between 0.6 and 0.9, with rwTTD being the most consistent endpoint. Real world endpoints were also consistent between institutions. At 1-year, rwOS ranged between 40 and 57%; these results were within the range of the median OS values from published clinical trials (69, 70). Two key conclusions were determined from this study: (1) real world endpoints are valid and should be used to support regulatory and payer decision-making; and (2) observed differences likely reflected true differences between real world and clinical trial populations and practices. Being conscious of these differences can inform risk mitigation for downstream clinical development and post-market surveillance.

As pharmacogenomics practice expands, the relative frequency of pharmacogene variants (drug-gene interactions), polypharmacy permutations (drug–drug interactions), and correlations related to drug- class adverse reactions might inform rare adverse effects. These toxicities, that are often difficult to detect within an RCT, might not be identified prior to FDA approval. Several drugs have been withdrawn from the market despite compelling RCT results resulting in FDA approval. In some cases, patients suffered great harm due to rare pharmacogenomics or drug–drug interactions that might not be previously known (80). For example, the cardiovascular toxicities of rofecoxib are suspected as being attributable to one or a combination of polymorphisms in a number of genes: 372 UGT2B7; (81) UGT2B15; (81) PTGS1; (12) CRP; (12) and PTGIR (82).

Combinations of rare but clinically consequential genotypes could easily be omitted from representation in the treatment (and control) arm of an RCT, but an ECA with genotype data might lend comparative insight before adverse events arise in the general population post FDA approval. The Observational Health Data Sciences and Informatics dataset contains 250 million cases. Within the dataset, 10% of diabetes, 24% of hypertension, and 11% of depression patients had unique treatment pathways (81). This then yields a daunting number of permutations in drug combinations that might materially impact safety or efficacy outcomes in RCTs. Compared to traditional RCT control arms, ECAs can validate the degree of similarity to the general population and identify factors for prospective pharmacovigilance later in drug development or during post market monitoring. RCTs are often considered to be an impractical means to validate clinical decision- making given an increasing number of pharmacogenomics variants (83), and that RWD is an important source of evidence in support of advancing the field of pharmacogenomics (50). Treating many rare diseases, oncology practice has been significantly influenced by RWD from registries and clinicogenomics databases for clinical decision support (84).

While variability exists within RCTs, variability also exists with ECAs. There are 3 types of ECAs which are accepted by regulatory agencies and based on time of cohort acquisition. Burcu and colleagues (64) characterized the 3 types of ECAs as historical, hybrid, and contemporaneous. Using their definition, historical ECAs almost exclusively use retrospective data, while the ECAs designated as hybrid and contemporaneous collect data both retrospectively and prospectively. A hybrid ECA, collecting both retrospective and prospective data, is often used to augment a control arm in an RCT. Hybrid ECAs are established either at the end of the single-arm trial using retrospective data from the time frame that the clinical trial was conducted, and the start of a single- arm trial with real-time follow-up. Contemporaneous ECAs generally provide a matched cohort from RWD for the control arm during the conduct of the clinical trial (85).

The control arms of RCTs seek to match the experimental arm patient demographics and comorbidities, and both arms are enrolled in the study at the same time. Most RCTs, however, do not match socioeconomic factors, such as the type of healthcare insurance payer, the type of health system, or the region of the country where care is delivered. Socioeconomic factors influence health behaviors, healthcare practice patterns, availability of medications and procedures, healthcare resource utilization (HCRU), and costs. RCTs conducted in a single-institution are poised to provide the least patient variability within the study, including HCRU, costs, and other socioeconomic factors, with all patients likely receiving treatment from the same facilities and health system procedures. In contrast, multi-institutional RCTs do not usually account for socioeconomic, health system, and regional variables within the randomization schema. Cooperative group trials rarely identify or statistically account for outlier institutions. Socioeconomic, health system and regional variables become more apparent when more diverse populations are included within a clinical trial or with RWD. Social determinants of health were highly evident during the COVID pandemic in which members of racial and ethnic minority groups, had higher rates of COVID-19 positivity, disease severity, and outcomes (86).

Defined by the 3 types of ECAs, historical, traditional, and contemporaneous, outcomes are dependent on expanded criteria that encompass the socioeconomic variables of a diverse RWE population (Table 2) (54). All three types of ECAs match the individual patient demographics and comorbidities for both the experimental and control arms within a RCT. However, regional variability can reflect access to care, rural vs. urban, academic medical center vs. community hospital or clinic. Most important to outcomes, however, are social determinants of health, including the type of health insurance, race, ethnicity and socioeconomic status.

**Table 2:** Comparison of control groups in randomized controlled trials (RCT) by single institution, multi-institution, traditional external control groups, and contemporaneous external control groups.

	RCT Control groups		External control groups		
	Single institution	Multi-institution	Historical	Traditional	Contempor aneous
Individual patient demographics	Y	Y	Y	Y	Y
Individual patient comorbidities	Y	Y	Y	Y	Y
Enrolled at the same time as the experimental arm	Y	Y	N	N	Y
Type of health insurance/Payer	P	N	N	P	Y
Health systems matched	Y	N	N	P	Y
Region matched^1^	Y	N	N	P	Y

Y represents-yes; N represents-no; P represents-perhaps. ^1^Regions within the United States: NorthEast/New England [ME, NH, VT, MA, CT, RI], NorthEast/Middle Atlantic [NY, PA, NJ], South/South Atlantic [WV, MD, DE, DC, VA, NC, SC, GA, FL], South/East South Central [KY, TN, AL, MS], South/West South Central [AR, LA, TX, OK], MidWest/East North Central [Mi, Wi, IL, IN, OH], MidWest/West North Central [MN, IA, MO, ND, SD, NE, KS], West/Mountain [MT, ID, WY, NV, UT, CO, AZ, NM], West/Pacific [WA, OR, CA, AK, HI].

Patients enrolled on an RCT generally have more access to care and are treated within a healthcare institution or system. Historical ECAs are either used for context as the standard of care for a selected time-period, or are retrospectively matched to the enrolled patients upon completion of a RCT. Especially when derived from datasets that are socioeconomically diverse and that include underserved populations, historical ECAs may not be an accurate cohort for the treatment arm of a clinical trial. In a large study, such as a multi-institutional RCT, the variances among institutions may not be significant enough to impact outcomes or compromise statistical significance. Most multi-institutional studies are conducted within academic medical centers or private institutes that foster medical research. In most cases, there is little difference among the variables associated with socioeconomic determinants of health in multi-institutional RCTs.

Much like the hybrid ECA, the demographics of patients within a traditional ECAs are matched during an interim analysis or at the end of the RCT. The traditional ECA is actively included within the study methods. Depending on the RCT population, the dataset from which the traditional ECA is derived can be specifically selected to include socioeconomic factors in the dataset. The contemporaneous ECA is more granular than the traditional ECA, and is the only control group that can match all the confounding variables of demographics, comorbidities, enrolled at the same time as the experimental arm patient, type of healthcare insurance, type of healthcare system delivery and access to care, and regional parameters. Using a contemporaneous ECA, theoretically, could yield the most exact comparison to the experimental arm of a RCT.

## 4. Discussion of limitations

The randomized, controlled prospective trial is the gold standard in rigorous science for several reasons. Prospective randomization of patients to a standard of care control and highly structured experimental protocol mitigates (but does not always eliminate) bias and enables for control of certain variables (but not all variables) that might confound a treatment effect (87). One study of RCT trials underlying 143 cancer drug approvals, 17% had suboptimal control arms (87). It can be argued that the use of genomic biomarkers can bias a trial by design in that patient selection strategies are geared toward a more specific hypothesis, so alignment of the hypothesis and study design with methodology is important to mitigate the inherent biases these tools can introduce (88).

Depending on how heterogenous a population is and whether a powered trial can be fully accessioned, a prospective, randomized controlled trial may not be feasible, cost-effective, or practical. For example, the use of pharmacogenomics to guide clopidogrel use is complex. Several biotransformation mechanisms are involved in the pharmacology of clopidogrel: CPY2C19 (1st step), CYP3A4 (2nd step), and CYP2C9 (89), implicating several hundred currently known variants of these three pharmacogenes. Clopidogrel is used for a number for different cardiovascular indications. Commercial genotyping of these pharmacogenes is currently limited to a small number of the most prevalent variants in commercially available tests. Thus, the RCTs that have been conducted for pharmacogenomic-guided clopidogrel use are very narrow use cases involving a single indication and a limited number of variants. The costs of validating each drug-pharmacogene-disease triad permutation of pharmacogenomic-guided use of clopidogrel in an RCT across so many domains would be prohibitive. The use of real-world clinical outcome data from an ECA (e.g., an un genotyped approach) can serve as a useful, albeit imperfect, comparator to assess utility of pharmacogenomic- guided use of drugs like clopidogrel across many domains, however, limited outcome data can itself result in biases (90). This is a more pragmatic approach to pilot and assess RCT feasibility or as an alternative form of evidence, by enabling the use of existing data instead of the costs of enrolling a participating control cohort. Pooling data is an approach that has been used confront sample size challenges in single health systems but data governance barriers across organizational barriers make this approach laborious (49, 51, 52).

There are limitations to the utility of ECAs, that vary depending on disease, clinical specialty, and importantly, the outcomes of interest. Sometimes, the clinical outcomes measures may not be in the EMR or measured with research-robust methodologies, or data does not meet GXP (e.g., GCP, GLP, GMP) and CDISC standards for use in regulatory filings, in cases where FDA review of the clinical study is required. Even in instances where the ECA data comes from a clinical trial such as the CRBM model that created digital twins to augment the control group (66), data limitations can limit ECA cohort size. This challenge is especially acute in rare diseases and involving registry data. When using data from an EMR this limitation can arise if data access to multiple health systems is impractical to obtain. ECAs can be a useful clinical study strategy for comparative effectiveness, genomic medicine, and pilot studies where regulatory hurdles are less burdensome. Use of an ECA requires some capacity to account for and mitigate bias, which inherently requires a data set with detailed clinical annotation, in addition to the capacity to do subanalysis for age, sex, stage of disease and other major determinants of outcomes. Comparisons of the ECA to the treatment arm may not be feasible for all clinical important clinical outcomes as a result of informatics silos or medical practices (5). The entire case-level natural history of disease would be an idealized data source (49), but in reality all data sets have significant blindspots across time and geography (91). The ECA need not be an alternative to a prospective internal control of standard of care (i.e., limited to single arm trials), should a strong validation of external validity be justified, which could make sense for phase 2 trials used to assess the merit of a phase 3 or label expansion program. The FDA provides guidance on the use of external control arms in drug and biologic product development (92).

## 5. Conclusion

Research paradigms are changing, and the pace of change was accelerated by the realities of conducting clinical trials during the COVID-19 pandemic (93). While control arms within RCTs seek to match the demographic and comorbidities of the experimental arm cohort, randomization procedures rarely account for socioeconomic factors or broader differences in the clinical setting. Important factors include the type of healthcare insurance, healthcare delivery system, access to care, and regional variations. There is a growing body of literature showing that ECAs are a valuable approach to increasing efficiency, reducing patient risk, and accounting for social determinants of health. Various ECA approaches are necessary to help capture RWD in ways that maximize the knowledge gained when a study is conducted. Multiple contemporaneous control arms, that include the disparate social determinants of health, could theoretically be incorporated within a clinical trial to anticipate the real- world results of the new therapeutic in Phase IV analysis. More than any other type of control arm, contemporaneous ECAs allow a granular match with the experimental arm cohort and are likely to become a preferred approach in clinical research as data quality and annotation improve real-world practice. The production of high quality RWD in medical specialties such as pharmacogenomics and oncology have great promise to achieve validation and accelerate implementation of new innovations that can reduce the costs and duration of clinical trials and improve care, while illuminating causes of health disparities.

## Author contributions

NJ and MS conceived of the work. NJ and PS made significant contributions to the literature review, research, drafting and revising of the manuscript. KR, GU, LZ, MS, and MO provided critical input on revisions, content, and organization. All authors contributed to the article and approved the submitted version.

## Conflict of interest

The authors declare that the research was conducted in the absence of any commercial or financial relationships that could be construed as a potential conflict of interest.

## Publisher’s note

All claims expressed in this article are solely those of the authors and do not necessarily represent those of their affiliated organizations, or those of the publisher, the editors and the reviewers. Any product that may be evaluated in this article, or claim that may be made by its manufacturer, is not guaranteed or endorsed by the publisher.

## Abbreviations

ECA: external control arm
FDA: Food and Drug Administration
EMA: European Medicines Agency
RCT: randomized controlled trials
ICU: intensive care unit
OCEBM: Oxford Centre for Evidence-Based Medicine
VTE: venous thromboembolism
RWD: real-world data
CDRH: Center for Devices and Radiological Health
CBER: Center for Biologics Evaluation and Research
NGS: next-generation sequencing
SPSO: semi-parametric stochastic ordering
CRBM: Conditional Restricted Boltzmann Machine
CYP2C19: Cytochrome P450 2C19
CYP2D6: Cytochrome P450 2D6
rwTTD: real world time-to-treatment-discontinuation
rwOS: real world overall survival
UGT2B7: UDP glucuronosyltransferase family 2 member B7
UGT2B15: UDP glucuronosyltransferase family 2 member B15
PTGS1: prostaglandin endoperoxide synthase 1
CRP: C-reactive protein
PTGIR: Prostaglandin I2 receptor
HCRU: healthcare resource utilization
